# Supplementary material for: Cortisol Interaction with Aquaporin-2 Modulates Its Water Permeability: Perspectives for Non-Genomic Effects of Corticosteroids
Source: Int J Mol Sci. 2023 Jan 12;24(2):1499. doi: 10.3390/ijms24021499 (PMC9862916; doi:10.3390/ijms24021499)
Supplement: Supplementary file 1 [file ijms-24-01499-s001.zip › Supplementary Figure S1.doc]

Supplementary material

***Supplementary Figure S1. Interactions between cortisol molecules and AQP2 extra-cellular or intra-cellular surface.*** *From experimental setup 3 : Number of H-bonds between cortisol molecules of the extra-cellular compartment (in green) and of the intra-cellular compartment (in purple) with AQP2.*
